# A *Streptococcus pyogenes* DegV protein regulates the membrane lipid content and limits the formation of extracellular vesicles

**DOI:** 10.1371/journal.pone.0284402

**Published:** 2023-04-27

**Authors:** Clara Lambert, Thifaine Poullion, Qiufen Zhang, Alain Schmitt, Jean-Marc Masse, Karine Gloux, Claire Poyart, Agnès Fouet

**Affiliations:** 1 Université Paris Cité, Institut Cochin, INSERM U1016, CNRS UMR8104, Paris, France; 2 Micalis Institute, INRAE, AgroParisTech, Université Paris-Saclay, Jouy en Josas, France; 3 AP-HP Centre-Université Paris Cité, Paris, France; Universidade Estadual de Ponta Grossa, BRAZIL

## Abstract

Membranes contain lipids that are composed of fatty acids (FA) and a polar head. Membrane homeostasis is crucial for optimal bacterial growth and interaction with the environment. Bacteria synthesize their FAs via the FASII pathway. Gram-positive bacteria can incorporate exogenous FAs which need to be phosphorylated to become substrate of the lipid biosynthetic pathway. In many species including staphylococci, streptococci and enterococci, this phosphorylation is carried out by the Fak complex, which is composed of two subunits, FakA and FakB. FakA is the kinase. FakB proteins are members of the DegV family, proteins known to bind FAs. Two or three FakB types have been identified depending on the bacterial species and characterized by their affinity for saturated and/or unsaturated FAs. Some species such as *Streptococcus pyogenes*, which causes a wide variety of diseases ranging from mild non-invasive to severe invasive infections, possess an uncharacterized additional DegV protein. We identify here this DegV member as a fourth FakB protein, named FakB4. The *fakB4* gene is co-regulated with FASII genes suggesting an interaction with endogenous fatty acids. *fakB4* deletion has no impact on membrane phospholipid composition nor on the percentage of other major lipids. However, the *fakB4* mutant strain produced more lipids and more extracellular membrane vesicles than the wild-type strain. This suggests that FakB4 is involved in endogenous FA binding and controls FA storage or catabolism resulting in a limitation of extracellular FA release via membrane vesicles.

## Introduction

Cell membranes commonly comprise a lipid bilayer composed of a polar head and an apolar body constituted of fatty acids (FA). FA structure and length are decisive for membrane topology and properties such as fluidity, permeability and integrity. These features are crucial for the adaptation of bacteria to various environments [1]. Most bacteria synthesize FAs via the fatty acid synthesis pathway FASII. This pathway is not essential in the Gram-positive bacteria that use exogenous FAs (eFAs) to synthesize their lipids [2–5]. Incorporation of eFAs into the lipids of Gram-positive bacteria involve a FA kinase (Fak) complex that phosphorylates eFAs, yielding acyl-PO_4_. The acyl-PO_4_ is used by PlsY and PlsC for lipid synthesis or transformed in an acyl-ACP, which is used by PlsC or elongated via FASII [6]. The Fak complex is composed of two proteins; FakA provides the kinase activity and interacts with FakB proteins; FakB proteins are members of the DegV family [7, 8]. DegV proteins bind FA with high affinity indicating that DegV proteins may take part in lipid transport and in FA metabolic processes [9]. Whereas single *fakA* genes are found in Firmicutes, *degV* genes are multiple; two *fakB* have been identified in *Staphylococcus aureus*, three in *Streptococcus pneumoniae*, and four in *Streptococcus suis*, *Enterococcus faecalis* and *Enterococcus faecium* [7, 10–12]. In *S*. *aureus*, FakB1 and FakB2 bind exclusively saturated and preferentially unsaturated FAs, respectively. In *S*. *pneumoniae* FakB1, FakB2 and FakB3 bind saturated, mono-unsaturated and poly-unsaturated FAs, respectively. In *E*. *faecalis* FakB1 binds preferentially saturated FAs, whereas the three others bind unselectively FAs [11]. Although FakB1 and FakB2 are well conserved, the third FakB may be more related to *S*. *pneumoniae* FakB3 or to *S*. *suis* FakB4. In the case of enterococci, FakB1, FakB2 and FakB4 are present but the last FakB, termed FakB5, does not resemble the FakB3s [12]. The role of *S*. *suis* FakB4 could not be studied by a biochemical approach because it appeared as inclusion bodies *in vitro* [12]. *S*. *pyogenes*, a Gram-positive human pathogen responsible for more than 500,000 deaths annually worldwide [13], also possesses four *degV* genes, encoding the three characterized FakB proteins as well as a fourth uncharacterized DegV protein [12, 14].

To decipher the role of this fourth DegV protein, encoded by the gene M28_Spy1638, we compared amino acid sequence with the known FakB proteins and determined whether this gene is coregulated with the FASII genes [14]. We also constructed a *fakB4* mutant strain to investigate potential impacts on bacterial FA and lipid compositions. Finally, we characterized potential structural effects of the absence of FakB4.

## Results

### The *S*. *pyogenes* uncharacteized *degV* gene encodes a FakB protein and is controlled by FabT

**Fig 1:**
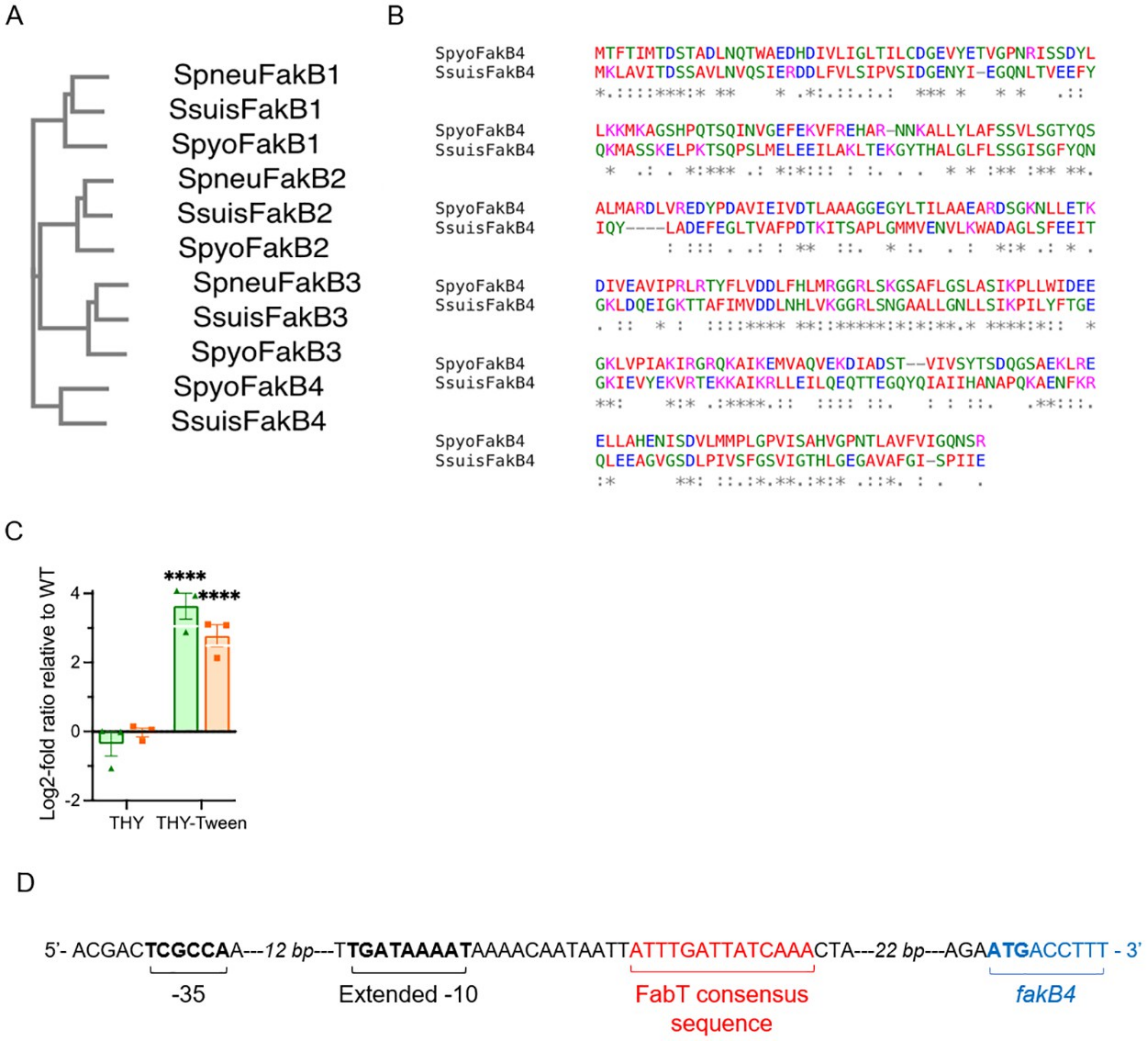
The M28_Spy1638 gene encodes a FakB4 protein and belongs to the FabT regulon. A) Phylogenetic tree of the three *S*. *pneumoniae* (Spneu), four *S*. *suis* (Ssuis), three *S*. *pyogenes* (Spyo), FakB proteins and the M28_Spy1638 product, named FakB4; the branch lengths are, from top to bottom, 0.17461, 0.15163, 0.20011, 0.13539, 0.13176, 0.17447, 0.15335, 0.1527, 0.18335, 0.22949, 0.21848. B) Sequence comparison of *S*. *pyogenes* and *S*. *suis* FakB4 proteins. C) Relative expression of *fabT*, green, and *fakB4* orange, in the mFabT versus the WT strain. Strains were grown in THY or THY-Tween 80 medium and RNAs were quantified by qRT-PCR. Expression was normalized to that of *gyrA*; relative gene expression is expressed as the log2-fold ratio in the mFabT *versus* the WT strain. 2-way ANOVA, Bonferroni post-test, ****p<0.0001. D) Cartoon representing the regulatory region of the *fakB4* gene with -35 and extended -10 boxes, previously published [18], FabT consensus sequence and *fakB4* translation initiation site indicated in black, red and blue, respectively.

In *S*. *pyogenes* M28PF1, the M28_Spy1638 gene product belongs to the DegV protein family. To determine whether it is a FakB protein and to which FakB protein it is most closely related, we constructed a phylogenetic tree using the FakB sequences from *S*. *pneumoniae* TIGR4 and *S*. *suis* 05ZYH33 (Fig 1A). The protein is most related to *S*. *suis* FakB4. The *S*. *pyogenes* DegV_1638_ protein has the same number of amino acid residues as the other FakB proteins, whereas *S*. *suis* FakB4 sequence lacks one fourth of the amino acid residues, from the 5^th^ to the 69^th^. Excluding the truncated sequence, these proteins share 58.62% identity. We consequently named *degV*_*1638*_ gene *fakB4* and the encoded protein FakB4. To give a clearer picture of the protein relatedness and the similarity between the all FakB proteins, we determined the identity percentages between all homologous FakB proteins (Table 1) and aligned the two FakB4 proteins (Fig 1B).

**Table 1:** Protein identity to *S*. *pyogenes* homologous FakB protein.

Species	FakB1		FakB2		FakB3		FakB4	
	Identity	Length	Identity	Length	Identity	Length	Identity	Length
*S*. *pyogenes*	100%	282	100%	283	100%	280	100%	286
*S*. *pneumoniae*	57.39%	282	63.89%	279	61.72%	281		
*S*. *suis*	61.94%	283	65.72%	277	61.46%	296	58.62%	222

M5005_Spy1650, the *fakB4* homolog in a M1 strain of *S*. *pyogenes*, is one of the six genes that is controlled by FabT, the FASII transcriptional repressor of the FASII genes, in all conditions tested [15]. FabT is present in streptococci, enterococci and lactococci. FabT belongs to the MarR family of regulators [for review, [16]]. The expression of the other *fakB* genes is not controlled by FabT in the M1 strain [15] and a FASII coregulation may shed light on the function of the protein. We therefore tested whether *fakB4* present in the M28PF1 strain is coregulated with FASII genes. We compared by qRT-PCR, the relative expressions of *fabT*, that is self-repressed, and *fakB4* in a *fabT* mutant strain (mFabT, S3 Table) *versus* in the WT strain. The mFabT strain expresses a FabT variant in which the histidine in position 105 was replaced by a tyrosine. The FabT^H105Y^ mutation leads to derepression of the FASII genes (to be published elsewhere), as previously described for a *S*. *pyogenes fabT* mutant strain [15]. FabT co-repressors are long chain acyl-Acyl Carrier proteins (acyl-ACP) [17]. Strains were therefore grown in THY and in THY-Tween 80; Tween 80 provides, the long chain unsaturated fatty acid 18:1Δ9 (Fig 1C). Neither *fabT* nor *fakB4* gene expression was affected by the FabT^H105Y^ mutation when the strains were grown in THY, the role of FabT being limited without the presence of the co-repressor. In contrast, *fabT* expression was derepressed in the mFabT strain in THY-Tween 80 (12.44-fold), indicating that the FabT^H105Y^ protein displays a hampered repression capacity. The expression of the *fakB4* gene was similarly affected by the *fabT* mutation in THY-Tween 80 (6.88-fold). We hypothesized that *fakB4* may be directly controlled by FabT. Consensus FabT binding sequences are found along the FASII locus, which includes *fabT* [[16], and references herein]. We sought whether one was also present downstream from the *fakB4* transcription start site previously described [18]. Using ANTTTGATTATCAAATT as a probe, we found the sequence, ATTTTGATTATCAAA, that shows 100% identity on 15 out of 17 nucleotides and that is localized between the transcription and the translation start sites (Fig 1B).

*fakB4* is therefore coregulated with the FASII genes in both *S*. *pyogenes* strains in which it has been studied and likely directly controlled by FabT. This suggests that, in contrast to FakB1, FakB2 and FakB3, FakB4 may not be involved in the incorporation of exogenous FA into the membrane lipids.

### FakB4 does not play a major role in exogenous FA incorporation

To further characterize the role of FakB4 we constructed an isogenic mutant strain in which *fakB4* is interrupted by the insertion of a plasmid containing a *fakB4* internal fragment (see Material and methods). We hypothesized that the growth characteristics of the WT and mFakB4 strain would be identical in rich laboratory medium, but may differ in FA-containing medium, if FakB4 was involved in specific FA uptake (S1 Fig). Both strains had exactly the same growth characteristics at 37 °C in THY, THY-Tween 80 or THY-FBS.

**Fig 2:**
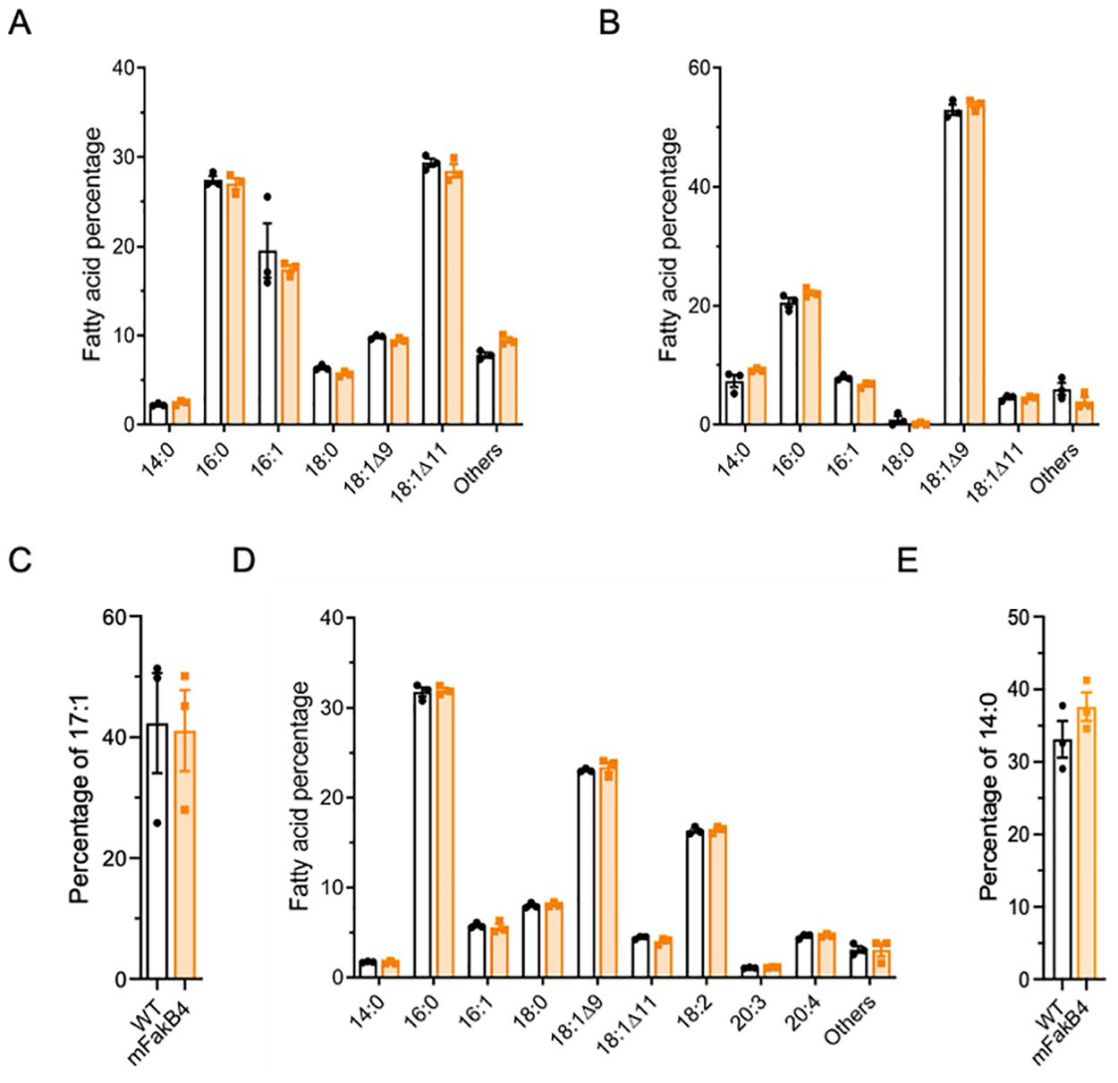
The WT and mFakB4 strains show identical relative FA compositions. A, B, D) Membrane fatty acid percentage of WT and mFakB4 strains grown in A) THY, B) THY-Tween 80, D) THY-Plasma; C, E) Membrane C) 17:1 or E) 14:0 percentage in WT and mFakB4 strains cultivated in THY-17:1 or C14:0; A-D, N = 3 (S1 Table). 2-way ANOVA, Bonferroni post-test.

The Fak complexes, containing the kinase FakA interacting with FakB isoforms, are responsible for eFA phosphorylation and transfer to the lipid synthesis pathway [7]. The deletion of given FakB proteins leads to default in the incorporation of specific FAs into the lipids. To test whether FakB4 is involved in eFA incorporation, we determined the composition of membrane lipids from mFakB4 and WT strains grown in various media (Fig 2A, 2B and 2D, S1 Table). The FA distribution was similar in WT and mFakB4 strains grown in THY (Fig 2A, S1 Table). The addition of Tween 80 strongly modified the FA distribution in the WT and the mFakB4 strains that remained similar; there was a strong diminution of 16:0 and 16:1 and a rise of 18:1Δ9, the major Tween 80 component, signing the eFA incorporation and FASII arrest (Fig 2B, S1 Table). This suggested that eFAs are similarly incorporated by both strains and that FakB4 is not required for 18:1Δ9 incorporation. A similar incorporation by both strains was checked by assessing the incorporation of 17:1, that is not produced by the bacteria (Fig 2C). The percentage in both strain membranes was the same confirming the absence of eFA incorporation defect in the mFakB4 strain. However, 17:1 and 18:1Δ9, provided by Tween 80, are monounsaturated FAs. We reasoned that if FakB4 was specific for other FAs, a difference may have been unnoticed. We consequently also determined the FA composition after growth in the presence of human plasma that also contains saturated and poly-unsaturated FAs (Fig 2D, S1 Table). Again, the membrane composition was different from those found in THY and THY-Tween 80 grown bacteria, but again similar in both strains. The concentration of poly-unsaturated 18:2 and 20:4 strongly rose, indicating that, as already shown for *Staphylococcus aureus*, *S*. *pneumoniae*, *S*. *agalactiae* and *E*. *faecalis*, *S*. *pyogenes* incorporated poly-unsaturated FAs [2, 3, 10, 19]. The saturated FA brought by plasma is 16:0, a FA that is synthesized by *S*. *pyogenes*, limiting the conclusion on *fakB4* impact on C16:0 incorporation. We thus also assessed exogenous 14:0 incorporation, a FA that is less produced by *S*. *pyogenes*. The percentage of exogenous C14:0 incorporated into membrane phospholipids is similar in both *fakB4* and WT strains (Fig 2E). These results indicate that the mFakB4 strain had no membrane FA composition defect whatever the FA environment tested and that FakB4 is not involved in eFA incorporation into the membrane phospholipids.

### FakB4 controls the lipid quantity

**Fig 3:**
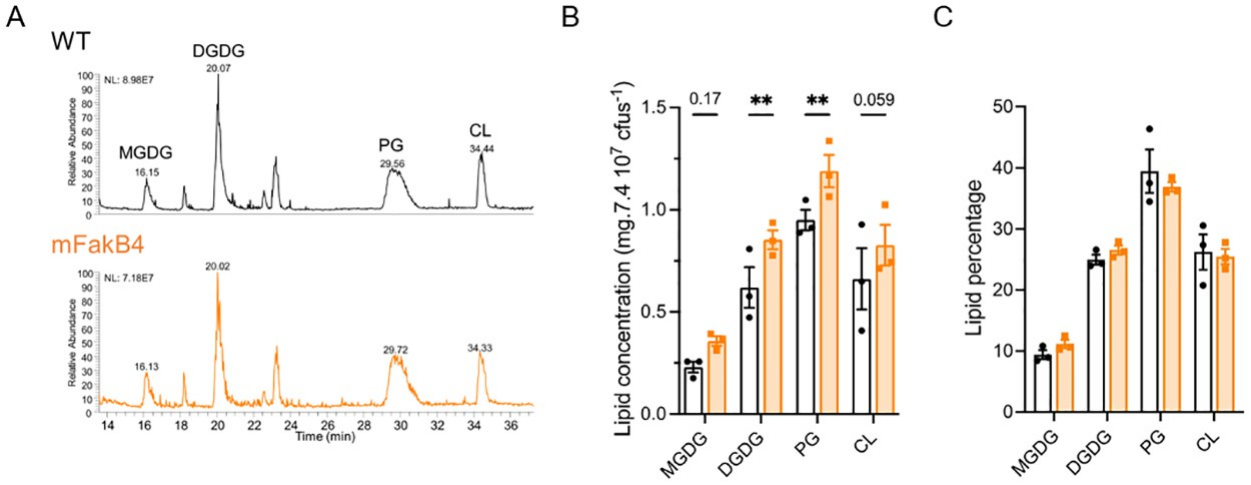
The WT and mFakB4 strains show different lipid quantities, but identical relative lipid composition. Lipid content in the WT and mFakB4 strains grown in THY, by class of lipids: A-B) lipid composition; A) HPLC-MS profile representing the main lipid classes, B) lipid concentration, C) ratio of lipid classes; N = 3 (S2 Table). Bars and symbols, white, WT strain; orange, mFakB4 strain; MGDG, monogalactosyldiacylglycerol; DGDG, digalactosyldiacylglycerol; PG, phosphatidylglycerol; CL, cardiolipin. 2-way ANOVA, Bonferroni post-test, **p<0.01; p values are indicated when between 0.05 <p<0.2.

The FA distribution being unmodified in the mFakB4 strain, we analyzed the global lipid contents in the strains (Fig 3A–3C, S2 Table). Contrary to FA analysis that yields an FA distribution, the global lipid analysis is normalized per cfu (Fig 3A and 3B). The digalactosyldiacylglycerol and phosphatidylglycerol concentrations per cfus were higher in the mFakB4 than in the WT strain and there was a trend to similar increases for the monogalactosyldiacylglycerol and the cardiolipins. Importantly, the ratios between the different lipid classes were, like those of the FAs, identical between both strains (Fig 3C, S2 Table). This suggests that the absence of FakB4 has a global impact on the four lipid families. We controlled that the lipid excess in the mFakB4 strain was not due to bacterial cell lysis that would produce cellular debris, modifying the OD/cfu ratio by determining that ratio during growth (S2 Fig). There was no difference in this ratio between the strains during growth. Altogether, these results indicated that the absence of FakB4 did not affect FA or lipid distribution but led to an increased lipid quantity per bacterial unit.

### The FakB4 strain expels extracellular vesicles

**Fig 4:**
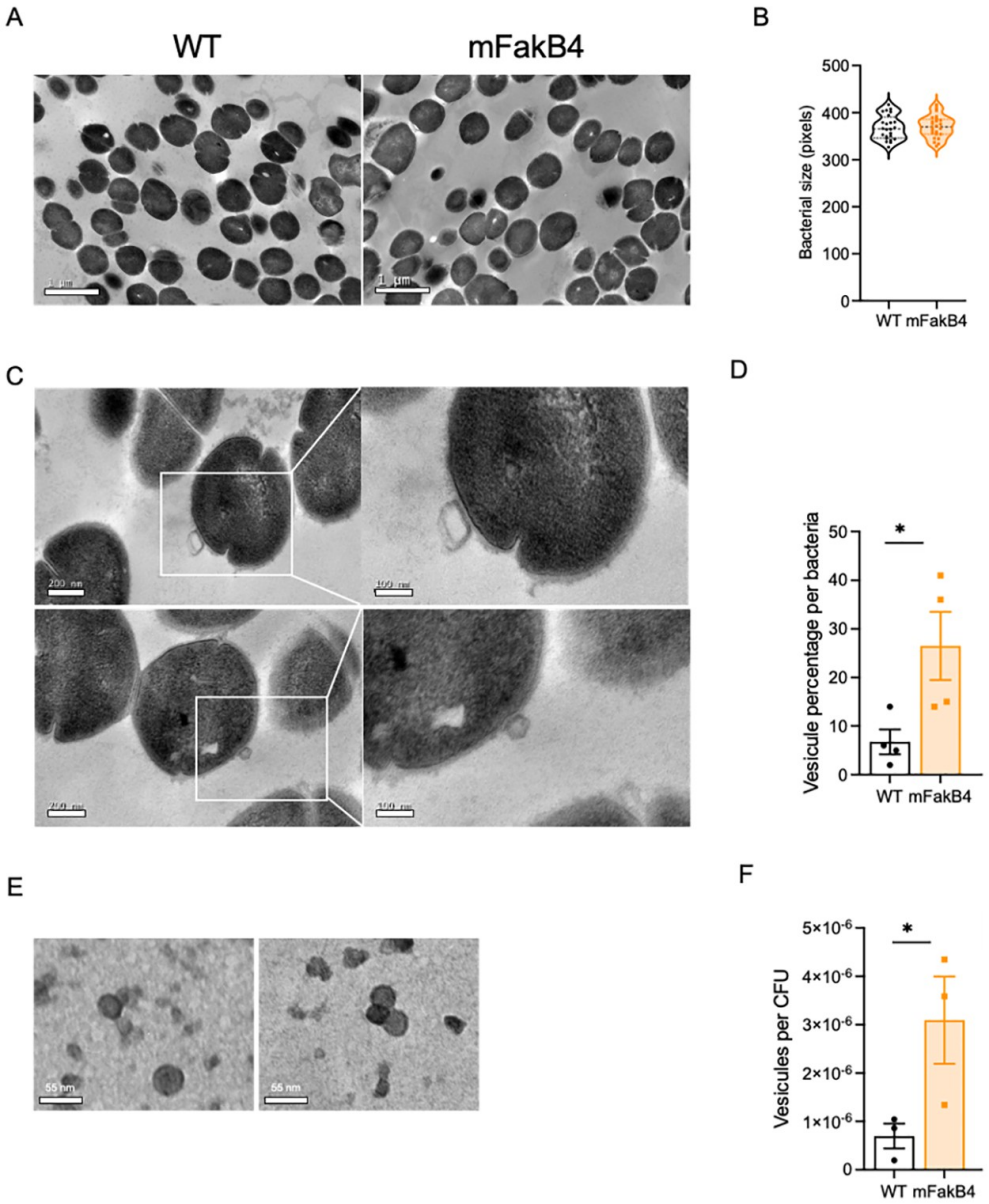
The mFakB4 strain secretes extracellular vesicles. A, C) TEM images of bacterial sections, left WT, right mFakB4; A) 4000X, C) 15000X, inset 30000X (scale = 200 nm; inset scale = 100 nm); B, D) Image analysis, B) bacterial diameter length, N = 4, 24 independent images, D) percentage of vesicles per bacteria, N = 4, 67 independent images. E) TEM images, of extracellular membrane vesicles contained in mFakB4 culture supernatant (scale = 40 nm); F) Image analysis N = 3, 30 independent images. B, D, F) One-way ANOVA, Bonferroni post-test, *p<0.05.

FA availability sets cell envelope capacity which in turn affects cell size [20]. We consequently hypothesized that the lipid increase observed in the mFakB4 strain may impact the bacterial morphology or the cell size. WT and mFakB4 strains grown in THY were consequently observed by transmission electron microscopy (TEM) and the diameter of bacteria measured (Fig 4A and 4B). Both strains displayed similar bacterial morphology and cell size. A close examination of the TEM images of the surface of *S*. *pyogenes* during the exponential phase indicated the presence of structures composed of double layers, extracellular membrane vesicles (EMV), particularly at the surface of the mFakB4 strain (Fig 4A). Their relative abundance in WT and mFakB4 strains was compared; there were 2.5-fold more EMVs at the surface of the mFakB4 strain than of the WT strain (Fig 4C and 4D, S3A Fig). These data suggest that extracellular vesicles attached to the bacterial surface contribute to the lipid surplus measured in the mFakB4 strain. We wondered whether EMVs could also be found in the culture supernatant and their abundance impacted by the *fakB4* mutation. Pelleted supernatants were therefore analyzed by TEM. EMVs were present and there were three times more EMVs in the supernatant from mFakB4 strain than in that of the WT strain (Fig 4E and 4F, S3B and S3C Fig). These data indicated that vesicle expulsion during the exponential phase of growth is increased in a *S*. *pyogenes* strain devoid of FakB4.

## Discussion

FakB proteins, members of the DegV family of proteins, bind eFAs and form a complex with FakA kinase that phosphorylate these FAs yielding substrates for PlsX or PlsY and further incorporation into lipids [6, 7, 10]. The specific roles of FakB1, FakB2 and, when present, FakB3, have been characterized in *S*. *aureus*, *S*. *pneumoniae* and *S*. *suis* [7, 10, 12]. However, some species possess a fourth DegV protein whose role has not been determined [12]. In the present work, we show that, in *S*. *pyogenes*, this fourth DegV protein is a FakB protein, which does not impact eFA incorporation in membrane lipids. The *fakB4* gene is coregulated with the FASII genes, and is therefore less expressed in the presence of eFAs than in their absence. This suggests that, whereas FakB1, FakB2 and FakB3 are involved in eFA incorporation, the role of FakB4 may be connected to endogenous FAs found in the cytosol [21].

The mFakB4 mutant strain produced more lipids than its wild-type counter-part. Microbial cell size is known to depend on FA availability [20]. Yet, the enhanced lipid abundance in the mFakB4 mutant strain did not result in an increased cell size but in an enhanced EMV production. This suggests that FakB4-FA binding disrupts this dependency. EMV production has been reported in *S*. *pyogenes* [22–25]. It is negatively regulated by the CovRS, a two-component system that controls the expression of 15% of *S*. *pyogenes* genes and that is turned down in response to sub-lethal concentrations of the antimicrobial peptide LL-37, a host produced peptide [22, 26]. In agreement with this regulation, extracellular membrane vesicles production is triggered by LL-37 [23]. This indicates that EMV production is stimulated in the host, where *S*. *pyogenes* is in the presence of eFAs. EMV production may be a FA detoxification mechanism. In *S*. *pyogenes*, EMVs are more abundant during late exponential or early stationary growth phase [22]. In contrast, in the *fakB4* mutant strain, the EMVs were more abundant than in the WT already in mid-exponential phase, in laboratory growth medium. This suggests that FakB4 limits EMV production during exponential phase through endogenous FA-binding and that EMV production likely compensates excess endogenous FAs toxicity.

The mFakB4 higher lipid production indicates that more acyl molecules are available in a *fakB4* mutant than in the wild-type strain. This strongly supports that, like the other members of the DegV family, FakB4 binds acyl molecules. It further indicates that, in contrast to FakB1—, FakB2- and FakB3-bound acyl molecules, the FakB4-bound acyl moieties are not subsequently transferred to the phospholipid biosynthetic pathway. Thus, FakB4 may have an important role in protecting the bacteria from FA excess. This can be carried out by two mechanisms. FakB4, by binding the supernumerary FA molecules, stores them. Alternatively, when FA molecules are in excess, FakB4 steers them towards a catabolic pathway that needs to be further characterized. Various FA detoxification mechanisms have been described [for review, [27]]. Among them, β-oxidation counteracts excess FAs in many bacteria. However, *S*. *aureus* that is unable to carry out this reaction possesses FarE, an efflux pump, which displays a detoxification function [28]. None of these mechanisms have been described in streptococci. *S*. *pyogenes* possesses a hydratase, the myosin cross-reactive antigen, that catalyzes the hydration of *cis*-9 and *cis*-12 double bonds [29]. Its gene is overexpressed in a *fabT* mutant strain in conditions that yield membrane composition modifications [15]. The hydration of unsaturated FAs prevents their accumulation which is detrimental to the bacteria. However, this mechanism produces saturated FAs, which may thus accumulate inside the bacterium and be toxic. FakB4, by taking care of these FAs, annihilate the toxicity without requiring their export.

Altogether this study indicates that the activities of FakB4 on the one hand, and of the three other FakB proteins, on the other, are entailed in different growth conditions; thus, these proteins target FAs from different origins and exhibit different roles. Further investigations on the FakB4 interactome will shed light on *S*. *pyogenes* lipid metabolism.

## Materials and methods

### Bacterial strains and culture conditions

The strains used in this study are described in S3 Table. *S*. *pyogenes* strains were grown under static condition at 37 °C in Todd Hewitt broth supplemented with 0.2% Yeast Extract (THY) or on THY agar plates. To study, the role of eFA addition, the medium was supplemented with 0.1% Tween 80 (THY-Tween 80) (Sigma-Aldrich, P1754), essentially composed of 18:1Δ9, with 100 μM 17:1 (Larodan, Sweden), with 10% Fetal Bovine Serum (THY-FBS), essentially composed of, approx., 43% saturated, 23% mono-unsaturated and 8% polyunsaturated FAs (Gibco), or with 10% human plasma (Etablissement Français du Sang) (THY-Plasma). For all experiments, strains were prepared as follows. Overnight cultures were diluted to an OD_600nm_ = 0.05 and grown in THY to the exponential phase (OD_600nm_ comprised between 0.4 and 0.5). The mFakB4 strain was grown in the presence of 10 μg.ml^-1^ erythromycin. *E*. *coli* strains were grown in LB or on LB agar plates supplemented with 150 μg.ml^-1^ erythromycin when appropriate.

### In silico analysis

*S*. *pyogenes* FakB protein sequences were extracted from the *S*. *pyogenes* M28PF1 strain, named WT in this study, using the Geneious software, searching for “DegV”. The FakB sequences of the *S*. *pneumoniae* TIGR4 strain (WP_000762061, WP_000161399, WP_000219939) and *S*. *suis* 05ZYH33 (SSU05_0827, SSU05_1650, SSU05_0561, SSU05_1899) were collected from NCBI. Sequence alignments and the phylogenetic tree, a Neighbour-joining tree without distance corrections were produced using Clustal W (1.83) multiple sequence alignment in EBI tools (https://www.ebi.ac.uk).

The presence of putative FabT binding sequences on the M28PF1 genome was sought with the ANTTTGATTATCAAATT sequence as a probe, accepting up to 2 mismatches using Geneious prime Biomatters development, www.geneious.com [16].

### RNA isolation

*S*. *pyogenes* strains were cultured at 37 °C in THY or THY-Tween 80, and cells were harvested at exponential growth phase (OD_600_ comprised between 0.4 and 0.5). After adding 2 volumes of RNA protect* (Qiagen), total RNA was extracted as previously described [30]. The bacteria from three independent cultures for each condition were lyzed by a 15 mg.ml^-1^ lysozyme, 300 U.ml^-1^ mutanolysine treatment for 30 min at 20 °C followed by two cycles of Fast-prep (power 6, 30 s) at 4 °C, then using the Macherey-Nagel RNA extraction kit, as indicated by the supplier. RNA integrity was analyzed using an Agilent Bioanalyzer (Agilent Biotechnologies) and a PCR. Absence of DNA was further confirmed by PCR.

### First-strand cDNA synthesis, quantitative PCR qPCR

Five hundred nanograms of total RNA was used for first-strand cDNA synthesis using SuperScriptTM II reverse transcriptase and random primers according to the manufacturer’s instructions (Invitrogen, Life technologies).

Quantitative PCR was carried out with SYBR Green PCR kits (Applied Biosystems, Life technologies) using four pairs of primers (S4 Table). *gyrA* and *rpoB* were used as the housekeeping reference genes. Relative quantification of specific gene expression was calculated with the 2−ΔΔCt method using *gyrA* as the reference gene and expressed in log2-fold change. Each assay was performed in triplicate on each sample as previously described [30].

### Strain construction

The primers used for the generation of the plasmids and verifying the different strains are described in S4 Table. The mFabT strain harbors a point mutation in *fabT*; the mFakB4 strain corresponds to an insertion interrupting the *fakB4* gene. They were obtained by homologous recombination of the plasmid pG1-mFabT or single cross-over with the plasmid pG1-DegVint2, respectively, following the same protocol as described previously [31, 32]. Regarding the *fabT* mutation, the plasmid pG1-mFabT was obtained by amplifying nucleotides -222 to +805, relative to the translation start site from the chromosome of a spontaneous mutant from our collection, CCH1963, where the C in position 313 was replaced by a T, leading to the replacement of the histidine in position 105 by a tyrosine. The strain was entirely sequenced as described previously, and no other mutation was found compared with the parent strain M28PF1 except for the point mutation in *fabT* [14]. For the mFakB4 strain, an internal fragment, from nucleotides 296 to 601 was amplified and cloned in pG1 using the In Fusion cloning kit^®^ (Clonetech) giving rise to the plasmid pG1-DegVint2. The plasmid was then inserted by a single cross-over event in M28PF1 chromosome, resulting in the interruption of *fakB4*. The absence of spurious mutations in the strains was verified by whole genome sequencing. This Whole Genome Shotgun project has been deposited at DDBJ/ENA/GenBank under the accession JAQMHW000000000. The version described in this paper is version JAQMHW010000000.

### Fatty acid analysis

Strains were grown in THY, THY-Tween 80, THY-Plasma or THY-17:1 until OD_600nm_ = 0.4–0.5. Fatty acids were extracted and analyzed as previously described [2, 3, 5]. Briefly, analyses were performed in a split-splitless injection mode on an AutoSystem XL Gas Chromatograph (Perkin-Elmer) equipped with a ZB-Wax capillary column (30 m x 0.25 mm x 0.25 mm; Phenomenex, France). Data were recorded and analyzed by TotalChrom Workstation (Perkin-Elmer). FA peaks were detected between 12 and 40 min of elution, and identified by comparing to retention times of purified esterified FA standards (Mixture ME100, Larodan, Sweden). Results are shown as percent of specific FA as calculated from their proportions compared to total peak areas (TotalChrom Workstation; Perkin Elmer).

### Lipid analysis

Strains were grown in THY until OD_600nm_ = 0.4–0.5. The cultures were diluted to 7.4 10^7^ cfus.mL^-1^. Lipid extractions were performed as previously described [5, 33, 34]. Lipids were identified following the method previously described [35]. The lipids separation was realized by normal phase HPLC (U3000 ThermoFisher Scientific) using a Inertsil Si 5μm column (150 x 2.1 mm I.D.) from GL Sciences Inc (Tokyo, Japan). Lipids were quantified using a Corona-CAD Ultra and identified by mass-spectrometry negative ionization and MS^2^/MS^3^ fragmentation (LTQ-Orbitrap Velos Pro). The concentration of each lipid class was determined as previously described using as standards DGDG, 840524P-5MG; MGDG, 840523P-5MG; CL (heart CA), 840012P-25MG; PG (egg), 841138P-25MG [36]. Lipids spectra were analyzed on Xcalibur^™^ software (ThermoFisher Scientific, version 4.2.47).

### Bacterial morphology and size analysis

THY exponential phase cultures (0.4 < OD_600nm_ < 0.5) were centrifuged and the pellets resuspended in 1 ml phosphate buffer saline (PBS) twice and incubated 30 min at 4 °C with a fixative solution (4% paraformaldehyde and 2.5% glutaraldehyde). Bacteria were then washed 3 times in PBS and subsequently treated as previously described, with slight modifications [20]. Secondary fixation was performed with 1% osmium tetroxide in PBS for 1 h at room temperature, then bacteria were centrifuged, the pellets washed 3 times in H_2_O and further dehydrated with increasing concentrations of ethanol (70%, 90%, and 100% twice). After dehydration, pellets were infiltrated with sequential 3:1, 1:1, and 1:3 solutions of resin (Embed 812 kit; Electron Microscopy Sciences, Hatfield, PA) and 100% ethanol for 1 h separately at room temperature. Finally, the samples were incubated with propylene oxide for 2 min and placed in the embedding molds with resin. The embedding resin blocks were polymerized in an oven at 60 °C for 24 h. Ultrathin sections (90 nm) were cut from the block surface using an ultramicrotome (Reichert Ultracut S ultramicrotome, Leica Microsystems) then stained with uranyl acetate and lead citrate (LFG, France) and observed by TEM (JEOL 1011, JEOL, Japan)) at 80 kV of acceleration voltage. Acquisitions were performed by an Orius 1000 CCD Camera (GATAN, USA) using Digital Micrograph software (GATAN, USA).

To determine the bacterial size, the larger bacteria from each independent image were selected as representative of those sliced through the sphere diameter. The pixels along the diameter were then quantified.

### Extracellular vesicle isolation and quantification

Extracellular vesicles were isolated from *S*. *pyogenes* strains cultured at 37 °C in THY as described previously [22]. PBS-washed pellets were further processed as follows. Small sample droplets (20 μl) were deposited on 200 mesh formvar coated copper grids for 2 min. The excess of sample solution was removed by blotting the grid with filter paper (Whatman). Grids were incubated with a drop of staining solution (uranyle acetate) for 2 min and blotted with a filter paper. The grid was deposited on a filter paper. Samples were immediately examined in a JEOL 1011 transmission electron microscope (JEOL, Japan) with an ORIUS 1000 CCD camera (GATAN, France), operated with Digital Micrograph software (GATAN, France) for acquisition.

## Supporting information

S1 Fig Influence of a *fakB4* mutation on bacterial growth in rich media. Wild-type, orange, and mFakB4, black, strains were precultured to mid-exponenetial phase, OD_600_ = 0.5 in THY and then diluted to on OD_600_ of 0.05 in A) THY, B) THY-Tween 80 and C) THY-fetal bovine serum. Growth was monitored in a Multiscan (Thermo Scientific). Note that the OD in the Mutliscan is roughly half of that in a classical spectrometer.
(DOCX)

S2 Fig Ratio CFU—OD_600_. WT and mFakB4 strains were grown in THY and samples were taken at different OD_600_ during growth; serial dilutions were plated. cfus were counted after incubating the plates 24 h at 37°C.
(DOCX)

S3 Fig The mFakB4 strain expels membrane vesicles; they are found attached to the bacteria and in the supernatant. A) TEM images of mFakB4 bacterial sections white arrows, vesicles (scale = 200 nm); B-C) TEM images of extracellular membrane vesicles (EMVs). B) Comparison of WT and mFakB4 culture supernatants (scale = 100 nm). C) Observation of EMVs contained in mFakB4 culture supernatant (scale = 55 nm); 15000X.
(DOCX)

S1 Table Consequences of a *fakB4* mutation on fatty acid membrane composition in different media. (DOCX)

S2 Table Membrane lipid family composition and proportion in WT and mFakB4 strains. (DOCX)

S3 Table Strains and plasmids used in this study. (DOCX)

S4 Table Primers used in this study for plasmid and strain construction and for PCR experiments. (DOCX)
