# Supplementary material for: A Streptococcus pyogenes DegV protein regulates the membrane lipid content and limits the formation of extracellular vesicles
Source: PLoS One. 2023 Apr 27;18(4):e0284402. doi: 10.1371/journal.pone.0284402 (PMC10138225; doi:10.1371/journal.pone.0284402)
Supplement: S1 Fig — Wild-type, orange, and mFakB4, black, strains were precultured to mid-exponenetial phase, OD600 = 0.5 in THY and then diluted to on OD600 of 0.05 in A) THY, B) THY-Tween 80 and C) THY-fetal bovine serum. Growth was monitored in a Multiscan (Thermo Scientific). Note that the OD in the Mutliscan is roughly half of that in a classical spectrometer. (DOCX) [file pone.0284402.s001.docx]

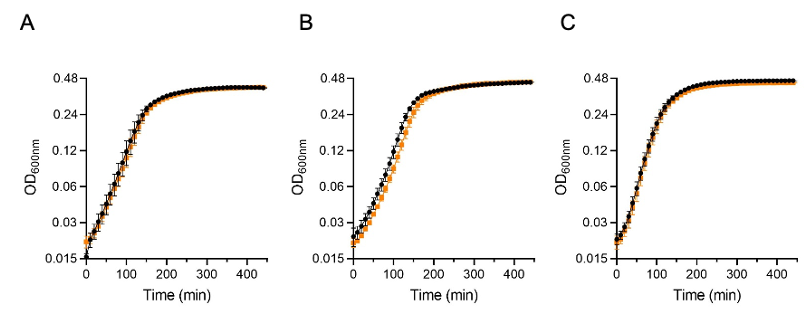


**Figure S1. Influence of a *fakB4* mutation on bacterial growth in rich media.** Wild-type, orange, and mFakB4, black, strains were precultured to mid-exponenetial phase, OD_600_ = 0.5 in THY and then diluted to on OD_600_ of 0.05 in A) THY, B) THY-Tween 80 and C) THY-fetal bovine serum. Growth was monitored in a Multiscan (Thermo Scientific). Note that the OD in the Mutliscan is roughly half of that in a classical spectrometer.
