# Supplementary material for: A Streptococcus pyogenes DegV protein regulates the membrane lipid content and limits the formation of extracellular vesicles
Source: PLoS One. 2023 Apr 27;18(4):e0284402. doi: 10.1371/journal.pone.0284402 (PMC10138225; doi:10.1371/journal.pone.0284402)
Supplement: S2 Fig — WT and mFakB4 strains were grown in THY and samples were taken at different OD600 during growth; serial dilutions were plated. cfus were counted after incubating the plates 24 h at 37°C. (DOCX) [file pone.0284402.s002.docx]

**
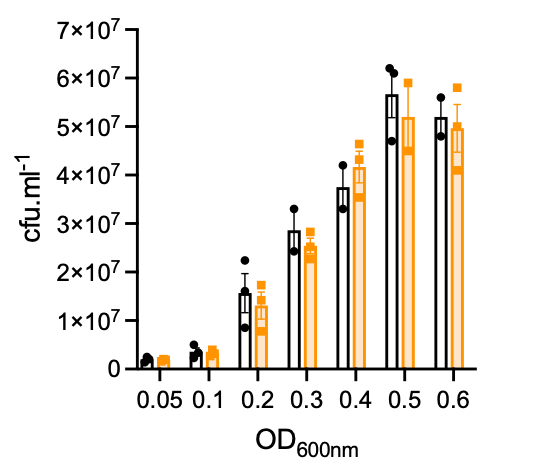
**

**Figure S2. Ratio CFU - OD_600_.** WT and mFakB4 strains were grown in THY and samples were taken at different OD_600_ during growth; serial dilutions were plated. cfus were counted after incubating the plates 24 h at 37°C.
