# Supplementary material for: A Streptococcus pyogenes DegV protein regulates the membrane lipid content and limits the formation of extracellular vesicles
Source: PLoS One. 2023 Apr 27;18(4):e0284402. doi: 10.1371/journal.pone.0284402 (PMC10138225; doi:10.1371/journal.pone.0284402)
Supplement: S3 Fig — A) TEM images of mFakB4 bacterial sections white arrows, vesicles (scale = 200 nm); B-C) TEM images of extracellular membrane vesicles (EMVs). B) Comparison of WT and mFakB4 culture supernatants (scale = 100 nm). C) Observation of EMVs contained in mFakB4 culture supernatant (scale = 55 nm); 15000X. (DOCX) [file pone.0284402.s003.docx]

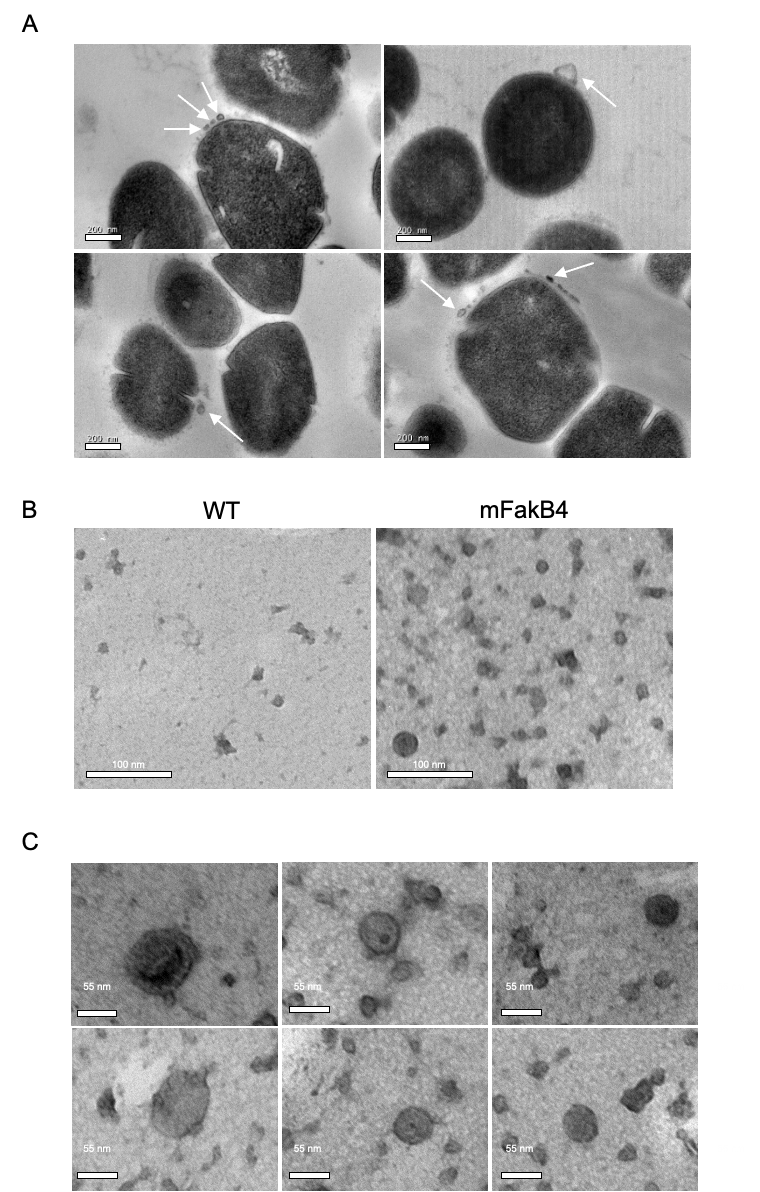


**Figure S3. The mFakB4 strain expels membrane vesicles; they are found attached to the bacteria and in the supernatant.** A) TEM images of mFakB4 bacterial sections white arrows, vesicles (scale = 200 nm); B-C) TEM images of extracellular membrane vesicles (EMVs). B) Comparison of WT and mFakB4 culture supernatants (scale = 100 nm). C) Observation of EMVs contained in mFakB4 culture supernatant (scale = 55 nm); 15000X.
