# Supplementary material for: A Streptococcus pyogenes DegV protein regulates the membrane lipid content and limits the formation of extracellular vesicles
Source: PLoS One. 2023 Apr 27;18(4):e0284402. doi: 10.1371/journal.pone.0284402 (PMC10138225; doi:10.1371/journal.pone.0284402)
Supplement: S1 Table — (DOCX) [file pone.0284402.s004.docx]

| **Table S1. Consequences of a *fakB4* mutation on fatty acid membrane composition in different media.** | | | | | | | | | | | | | | | |  |
| --- | --- | --- | --- | --- | --- | --- | --- | --- | --- | --- | --- | --- | --- | --- | --- | --- |
|  | THY | | | |  | THY-Tween | | | | | THY-Plasma | | | |  | Plasma |
| Strain | WT | | mFakB4 | |  | WT | | mFakB4 | |  | WT | | mFakB4 | |  |  |
| 14:0 ^a^ | 2.19 | ± 0.14 | 3.2 | ± 0.16 |  | 7.33 | ± 1.80 | 9.14 | ± 0.29 |  | 1.73 | ± 0.07 | 1.64 | ± 0.18 |  | 1.16 |
| 16:0 | 26.37 | ± 1.47 | 29.91 | ± 1.06 |  | 20.61 | ± 1.33 | 22.17 | ± 0.73 |  | 31.78 | ± 0.89 | 31.95 | ± 0.49 |  | 25.58 |
| 16:1 | 16.83 | ± 0.51 | 19.06 | ± 0.10 |  | 7.87 | ± 0.43 | 6.66 | ± 0.43 |  | 5.79 | ± 0.30 | 5.58 | ± 0.64 |  | 3.49 |
| 18:0 | 6.27 | ± 0.25 | 5.79 | ± 0.15 |  | 0.84 | ± 1.03 | 0.12 | ± 0.20 |  | 8.01 | ± 0.30 | 8.09 | ± 0.24 |  | 8.14 |
| 18:1∆9 | 9.9 | ± 0.21 | 8.8 | ± 0.07 |  | 52.95 | ± 1.47 | 53.76 | ± 0.94 |  | 23.06 | ± 0.20 | 23.43 | ± 0.96 |  | 22.09 |
| 18:1∆11 | 29.59 | ± 1.96 | 26.33 | ± 0.49 |  | 4.52 | ± 0.48 | 4.45 | ± 0.32 |  | 4.46 | ± 0.18 | 4.01 | ± 0.36 |  | 0 |
| 18:2 | 0 |  | 0 |  |  | 0 |  | 0 |  |  | 16.34 | ± 0.43 | 16.45 | ± 0.37 |  | 33.72 |
| 20:3 | 0 |  | 0 |  |  | 0 |  | 0 |  |  | 1.09 | ± 0.04 | 1.13 | ± 0.05 |  | 0 |
| 20:4 | 0 |  | 0 |  |  | 0 |  | 0 |  |  | 4.61 | ± 0.27 | 4.65 | ± 0.18 |  | 5.81 |
| Others ^b^ | 8.03 | ± 0.61 | 6.91 | ± 1.14 |  | 2.96 | ± 0.77 | 1.25 | ± 0.87 |  | 3.14 | ± 0.62 | 3.08 | ± 1.30 |  | 0 |
| (C16):(C18)^c^ | 0.94 |  | 1.2 |  |  | 0.49 |  | 0.49 |  |  | 0.72 |  | 0.72 |  |  | 0.45 |
| UFA:SFA ^d^ | 1.62 |  | 1.39 |  |  | 2.27 |  | 2.06 |  |  | 1.33 |  | 1.33 |  |  | 1.87 |
| ^a^ Percentage of total FAs | | |  |  |  |  |  |  |  |  |  |  |  |  |  |  |
| ^b^ Fatty acids representing less than 2 % total FAs | | | | |  |  |  |  |  |  |  |  |  |  |  |  |
| ^c^ Ratio of length chain = (C18:0 + C18:1)/(C16:0 + C16:1) | | | | | | | |  |  |  |  |  |  |  |  |  |
| ^d^ Ratio unsaturated/saturated FAs = (16:1 + 18:1∆9 + 18:1∆11)/(14:0 + 16:0 + 18:0) | | | | | | | | | | |  |  |  |  |  |  |

Strains were grown in THY, THY-Tween 80, THY-Plasma or THY-Plasma until OD_600nm_ = 0.4 - 0.5. Fatty acids were extracted and analyzed as previously described (Hays *et al*. J Bacteriol. 2021;203(20):e0022121. Epub 2021/07/27. doi: 10.1128/JB.00221-21.) Results are shown as percent of specific FA as calculated from their proportions compared to total peak areas (TotalChrom Workstation; Perkin Elmer). Mean values ± standard deviation of three independent experiments are shown.
