# Supplementary material for: A Streptococcus pyogenes DegV protein regulates the membrane lipid content and limits the formation of extracellular vesicles
Source: PLoS One. 2023 Apr 27;18(4):e0284402. doi: 10.1371/journal.pone.0284402 (PMC10138225; doi:10.1371/journal.pone.0284402)
Supplement: S2 Table — (DOCX) [file pone.0284402.s005.docx]

| **Table S2. Membrane lipid family composition and proportion in WT and mFakB4 strains.** | | | | | | | | | | |  |
| --- | --- | --- | --- | --- | --- | --- | --- | --- | --- | --- | --- |
| **Lipids** |  | **Lipid composition** | | | |  | **Lipid proportion** | | | |  |
|  |  | **WT** | | **mFakB4** | |  | **WT** | | **mFakB4** | |  |
| MGDG |  | 0.229 | ± 0.05 | 0.357 | ± 0.04 |  | 9.4% | ± 1.3 % | 11.1% | ± 1.2 % |  |
| DGDG |  | 0.619 | ± 0.17 | 0.853 | ± 0.08 |  | 25.0% | ± 1.4 % | 26.5% | ± 1.3 % |  |
| PG |  | 0.949 | ± 0.09 | 1.189 | ± 0.14 |  | 39.4% | ± 6.2 % | 36.9% | ± 1.3 % |  |
| CL |  | 0.661 | ± 0.26 | 0.827 | ± 0.17 |  | 26.2% | ± 5.0 % | 25.5% | ± 2.2 % |  |
| MGDG, monogalactosyldiacylglycerol; DGDG, digalactosyldiacylglycerol; PG, phosphatidylglycerol; CL, cardiolipin.  Strains were grown in THY until OD_600nm_ = 0.4 - 0.5. The cultures were diluted to 7.4 10^7^ cfus.mL^-1^ and lipid composition is expressed as mg/ 7.4 10^7^ cfus. Lipid extractions were performed as previously described (Kenanian G *et al*., Cell Rep. 2019;29(12):3974-82 e4. Epub 2019/12/19. doi: 10.1016/j.celrep.2019.11.071.) Lipids were identified following the method previously described (Abreu S et al., J Chromatogr A. 2017;1514:54-71. Epub 2017/08/05. doi: 10.1016/j.chroma.2017.07.063.). Lipids spectra were analyzed on Xcalibur^TM^ software (ThermoFisher Scientific, version 4.2.47). Mean values ± standard deviation of three independent experiments are shown. To establish the lipid proportion, each lipid composition value has been divided by the amount of lipid measured. | | | | | | | | | | |  |
|  |  |  |  |  |  |  |  |  |  |  |  |
|  |  |  |  |  |  |  |  |  |  |  |  |
