# Supplementary material for: A Streptococcus pyogenes DegV protein regulates the membrane lipid content and limits the formation of extracellular vesicles
Source: PLoS One. 2023 Apr 27;18(4):e0284402. doi: 10.1371/journal.pone.0284402 (PMC10138225; doi:10.1371/journal.pone.0284402)
Supplement: S3 Table — (DOCX) [file pone.0284402.s006.docx]

| **Table S3. Strains and plasmids used in this study.** | | |
| --- | --- | --- |
| **Strains or plasmids** | **Relevant properties** | **Source or references** |
| ***Streptococcus pyogenes*** | |  |
| WT | Wild-type representative *emm28* clinical isolate, M28PF1 | 1 |
| mFabT | M28PF1 mutated in *fabT* C313T (H105Y) | This study |
| mFakB4 | M28PF1 mutated in *fakB4*, interrupted by an insertion | This study |
| ***Escherichia coli*** | |  |
| Stellar^TM^ | F-, endA1, supE44, thi-1, recA1, gyrA96, phoA, Φ80d lacZΔ M15, Δ(lacZYA – argF) U169, Δ(mrr – hsdRMS –mcrBC), ΔmcrA, λ- | Clontech |
| **Plasmids** |  |  |
| pG1 | Thermosensitive broad-host-range vector | 2 |
| pG1-mFabT | pG1 containing the *fabT* gene with the point mutation C313T | This study |
| pG1-DegVint2 | pG1 containing an internal fragment of *fakB4* | This study |
| 1. Longo M, De Jode M, Plainvert C, Weckel A, Hua A, Chateau A, Glaser P, Poyart C, Fouet A. 2015. Complete Genome Sequence of *Streptococcus pyogenes emm28* Clinical Isolate M28PF1, Responsible for a Puerperal Fever. Genome Announc 3. | | |
| 2. Biswas I, Gruss A, Ehrlich SD, Maguin E. 1993. High-efficiency gene inactivation and replacement system for gram-positive bacteria. J Bacteriol 175:3628-35. | | |
