# Supplementary material for: A Streptococcus pyogenes DegV protein regulates the membrane lipid content and limits the formation of extracellular vesicles
Source: PLoS One. 2023 Apr 27;18(4):e0284402. doi: 10.1371/journal.pone.0284402 (PMC10138225; doi:10.1371/journal.pone.0284402)
Supplement: S4 Table — (DOCX) [file pone.0284402.s007.docx]

| **Table S4. Primers used in this study for plasmid and strain construction and for PCR experiments.** | | | |
| --- | --- | --- | --- |
| **Primer name** | **Sequence** | **Used for** |  |
| P47 | CGCCAGGGTTTTCCCAGTCACGAC | plasmid construction |  |
| RP48 | AGCGGATAACAATTTCACACAGGA | plasmid construction |  |
| FabT-222_am | GACTCTAGA**GGATCC**GGTCTGGCAAAGCTTTTTCA | pG1-mFabT construction |  |
| Fabt+805_av | CATGATTAC**GAATTC**GGCACCTGATGCAATAAGCT | pG1-mFabT construction |  |
| degVintF2 | CGACTCTAGA**GGATCC**GCGCTCTAATGGCACGCGAT | pG1-DegVint2 construction |  |
| degV intR2 | CCATGATTAC**GAATTCC**CTTCCTCATCAATCCACA | pG1-DegVint2 construction |  |
| ermB-F | GAGTGTGTTGATAGTGCAGT | mFakB4 strain construction |  |
| ermB-R | TAGGCGCTAGGGACCTCTTTA | mFakB4 strain construction |  |
| gyrA1 | CTGCTCGTATTACGGGTGATGT | qRT-PCR of *gyrA* |  |
| gyrA2 | GGCGATAACTCCACCACTGA | qRT-PCR of *gyrA* |  |
| rpoB1 | CCGTACACGTCGTAGCTTTT | qRT-PCR of *rpoB* |  |
| rpoB2 | GCATCGTGGATACGAGCTTC | qRT-PCR of *rpoB* |  |
| FabT-3’ | CCCTAGTCCACGCACCAATGCCT | qRT-PCR of *fabT* |  |
| FabT-5’ | GGGACAGTAACGACTAGCTTAAATA | qRT-PCR of *fabT* |  |
| 1638-Am | GCCAGCTTTCATCTTTTTCAAAAGG | qRT-PCR of *fakB4* |  |
| 1638-Av | GGGCAGAAGATCATGATATTGTC | qRT-PCR of *fakB4* |  |
| * in bold, added restriction enzyme sites, EcoRI or BamH1. | |  |  |
